# Resveratrol ameliorates fibrosis and inflammation in a mouse model of nonalcoholic steatohepatitis

**DOI:** 10.1038/srep22251

**Published:** 2016-02-25

**Authors:** Takaomi Kessoku, Kento Imajo, Yasushi Honda, Takayuki Kato, Yuji Ogawa, Wataru Tomeno, Shingo Kato, Hironori Mawatari, Koji Fujita, Masato Yoneda, Yoji Nagashima, Satoru Saito, Koichiro Wada, Atsushi Nakajima

**Affiliations:** 1 Department of Gastroenterology and Hepatology, Yokohama City University Graduate School of Medicine, 3-9 Fukuura, Kanazawa-ku, Yokohama, 236-0004, Japan; 2 Department of Surgical Pathology, Tokyo Women’s Medical University, 8-1, Kawada-cho, Shinjuku-ku, Tokyo 162-8666, Japan; 3 Department of Pharmacology, Shimane University Faculty of Medicine, 89-1 Enya-cho, Izumo, Shimane, 693-8501, Japan

## Abstract

The natural polyphenol compound resveratrol (RSV) is considered to have a broad spectrum of beneficial biological activities upon human health. However, the exact effect of RSV on steatosis (a phenotype of non-alcoholic fatty liver [NAFL]) or fibrosis and inflammation (major phenotypes of non-alcoholic steatohepatitis [NASH]) is not known. Our data showed that administration of RSV (2 or 20 mg/kg/day) did not suppress steatosis in a high-fat diet-induced model of NAFL in mice. In contrast, identical concentrations of RSV dramatically inhibited inflammation and fibrosis in a low-dose lipopolysaccharide-induced model of NASH. These data suggested that RSV administration-mediated improvement of inflammation and fibrosis was due to the inhibition of LPS reactivity controlled by CD14 expression in Kupffer cells. These findings suggest that RSV could be a candidate agent for the treatment of NASH.

Obesity is becoming increasing prevalent in the developed world, and can have a negative impact on certain types of chronic disease^1^,^2^,^3^. One of the most severe complications of obesity is non-alcoholic fatty liver disease (NAFLD). NAFLD comprises non-alcoholic fatty liver (NAFL) and non-alcoholic steatohepatitis (NASH). NAFL (which is characterised by steatosis) is considered to progress to a more severe type of liver disease associated with fibrosis and inflammation: NASH. NASH is thought to lead to liver cirrhosis and hepatocellular carcinoma, resulting in a decrease in quality of life and an increase in the risk of death^4^,^5^. The only effective treatment for NASH is weight loss by caloric restriction. However, preventing the progression of fibrosis and inflammation completely only by caloric restriction is difficult. Therefore, development of effective pharmacotherapy is required^6^,^7^.

Resveratrol (RSV) is a natural polyphenol compound considered to have a broad spectrum of beneficial biological activities upon human health. It has anti-inflammatory and anti-oxidant activities, which helps to protect the nervous system and cardiac system, and also is an anti-tumour agent^8^,^9^,^10^. In addition, it is expected to affect obesity-related complications by mimicking of calorie restriction^11^,^12^ *via* activation of key regulators of metabolic health, such as AMP-activated kinase (AMPK), nuclear factor (erythroid-derived)-like 2 (Nrf2), and nicotinamide adenine dinucleotide NAD+-dependent deacetylase (SIRT1)^13^. Moreover, studies have demonstrated the anti-steatosis effect of RSV using different models of liver steatosis^11^,^14^,^15^,^16^. However, the exact effect of RSV on steatosis (a phenotype of NAFL) or fibrosis and inflammation (major phenotypes of NASH) is not known.

We investigated if RSV administration improves steatosis, fibrosis or inflammation, or the pathogenesis of NAFL or NASH, in mouse models of these diseases.

## Results

### Effect of RSV on NAFL pathogenesis

**Figure 1:**
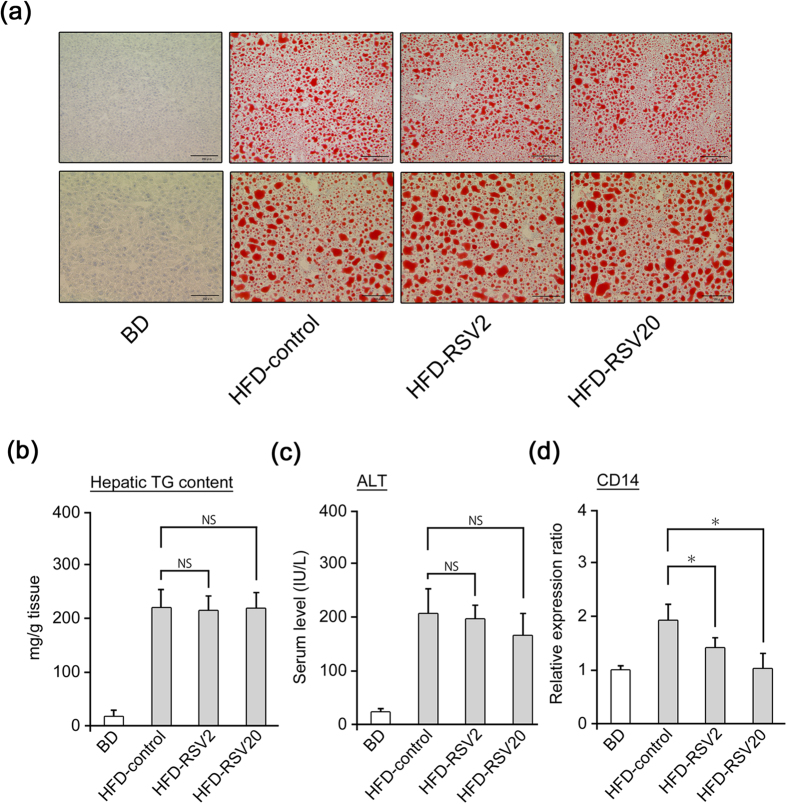
4-week administration of RSV does not reduce hepatic fat accumulation but RSV can inhibit mRNA levels of hepatic CD14. **(a–d)** Mice were separated into four groups: mice receiving a basal diet (BD) (*n* = 5, BD group); mice receiving a high-fat diet (HFD) (*n* = 5, HFD-control group); mice receiving a HFD mixed with RSV 2 mg/kg/day (*n* = 5, HFD-RSV2-treated group); mice receiving a HFD mixed with RSV 20 mg/kg/day (*n* = 5, HFD-RSV20-treated group). NAFL (steatosis) mouse model was created by feeding mice a HFD for 12 weeks and RSV was administered for the last 4 weeks **(a)** Oil Red O staining of liver tissue from mice of each group (first row: magnification ×100, scale bar: 200 μm; second row: magnification ×200, scale bar: 100 μm). Lipid droplets are stained red. **(b)** Measurement of accumulation of triglyceride (TG) in the liver from mice of each group (*n* = 5 per group). **(c)** Serum levels of alanine aminotransferase (ALT) in mice of each group. (*n* = 5 per group). **(d)** Expression of CD14 mRNA from mice of each group using quantitative real-time PCR. (*n* = 5 per group). Data represent relative expression compared with HFD-control group mice after normalization with expression of β-actin mRNA. Error bars denote mean ± standard deviation. Statistical significance was determined using the unpaired two-tailed Student’s *t*-test for parametric factors, and Mann–Whitney test for non-parametric factors. Asterisk denotes significant differences (*P < 0.05). n.s.: not significant.

For evaluation of the effects of RSV on NAFL pathogenesis (steatosis), the experimental design of this study is shown in Supplementary Fig. S1a,b. C57BL/6J mice were separated into four groups: (1) mice receiving a basal diet (BD) (*n* = 5, BD group); (2) mice receiving a high-fat diet (HFD) (*n* = 5, HFD-control group); (3) mice receiving a HFD mixed with RSV at 2 mg/kg/day (*n* = 5, HFD-RSV2-treated group); (4) mice receiving a HFD mixed with RSV at 20 mg/kg/day (*n* = 5, HFD-RSV20-treated group). Mice were fed a BD or HFD for 12 weeks, and RSV was administrated for the last 2 weeks (Supplementary Fig. S1a) or 4 weeks (Supplementary Fig. S1b). RSV preparation is described in the Methods section. During this period, steatosis in HFD-fed mice was also confirmed in histopathological observations by Oil Red O staining (Fig. 1a). Hepatic triglyceride contents were also increased in HFD-fed mice (Fig. 1b). In addition to steatosis, elevation of ALT levels was observed in HFD-fed mice (Fig. 1c). 4-week administration, as well as 2-week administration of RSV (Supplementary Fig. S2a–c and Supplementary Table S1) did not result in inhibition of lipid accumulation (Fig. 1a,b, HFD-control group *vs*. HFD-RSV2-treated group or HFD-RSV20-treated group) or elevation of ALT levels (Fig. 1c, HFD-control group *vs*. HFD-RSV2-treated group or HFD-RSV20-treated group). Intake of food or water among the three groups was not significantly different (Table 1). There were no significant differences in body weight or HOMA-IR among the three groups (Table 1). Marked effects were not observed for suppression of steatosis by 4-week administration of RSV compared with 2-week administration, so subsequent results are based on 4-week administration. To investigate the mechanism of action of RSV on steatosis, we undertook cDNA microarray analyses in the livers of the HFD-RSV20-treated group (*n* = 3) compared with HFD-control group (*n* = 3) (Supplementary Table S2) at 4 weeks of administration. However, no dramatic alterations in mRNA levels related to lipid metabolism (or other types of metabolism) were observed upon RSV treatment. These results suggested that RSV administration did not suppress steatosis development in an *in vivo* model of NAFL.

Surprisingly, hepatic expression of CD14 was inhibited significantly by RSV-treated (HFD-RSV2-treated group and HFD-RSV20-treated group) mice in comparison with HFD-fed mice (Fig. 1d, Supplementary Fig. S1d, Supplementary Table S2). CD14 expression is a marker of activation of Kuppfer cells in the liver (which is thought to cause inflammation and fibrosis in the liver^17^). Therefore, we focused on the effect of RSV on amelioration of inflammation and fibrosis.

### RSV attenuated hepatic inflammation through inhibition of the STAT3-CD14 pathway

Previously, we reported that CD14 overexpression induced by STAT3 signalling in the liver triggers NASH progression in our HFD fed-LPS-administered NASH model in mice^17^. To clarify the mechanisms of RSV-attenuated hepatic inflammation and fibrosis, we investigated the effect of RSV on the STAT3-CD14 pathway in a single LPS-administered model of inflammation. The experimental design is shown in Supplementary Fig. S1c. The NASH (inflammation) mouse model was created by feeding mice a HFD for 12 weeks in combination with administration of a single, low-dose of LPS (0.25 mg/kg/day) in accordance with our previous study^17^. Mice were divided into four groups: (1) mice receiving a BD in combination with LPS administration (*n* = 5, BD+LPS group); (2) mice receiving a HFD in combination with LPS administration (*n* = 5, HFD+LPS-control group); (3) mice receiving a HFD mixed with RSV2 in combination with LPS administration (*n* = 5, HFD+LPS-RSV2-treated group); (4) mice receiving a HFD mixed with RSV20 in combination with LPS administration (*n* = 5, HFD+LPS-RSV20-treated group).

**Figure 2:**
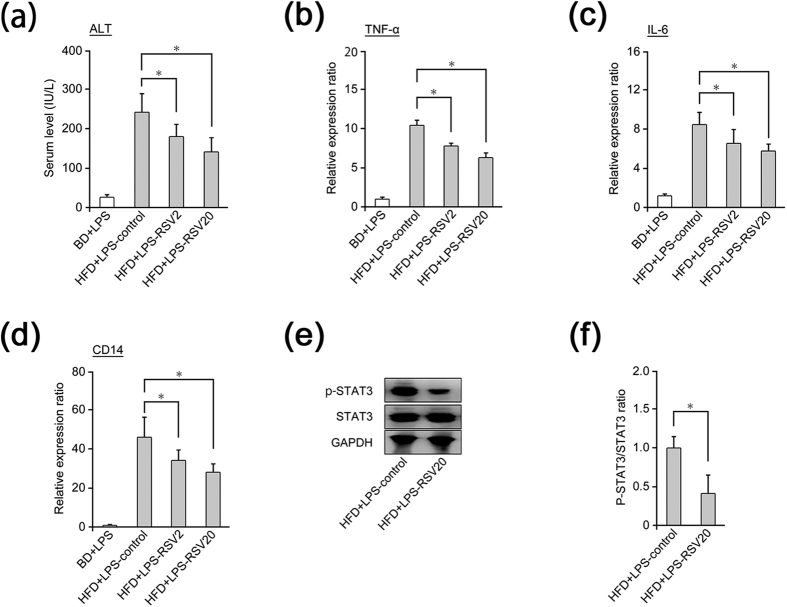
RSV attenuated single low-dose LPS-induced hepatic inflammation through inhibition of the STAT3-CD14 pathway. **(a**–**f)** Mice were divided into four groups: mice receiving a basal diet (BD) in combination with lipopolysaccharide (LPS) administration (*n* = 5, BD+LPS group); mice receiving a high-fat diet (HFD) in combination with LPS administration (*n* = 5, HFD+LPS-control group); mice receiving a HFD mixed with resveratrol (RSV) 2 mg/kg/day (RSV2) in combination with LPS administration (*n* = 5, HFD+LPS-RSV2-treated group); mice receiving a HFD mixed with RSV 20 mg/kg/day (RSV20) in combination with LPS administration (*n* = 5, HFD+LPS-RSV20-treated group). NASH (inflammation) mouse model was created by feeding mice a HFD for 12 weeks in combination with single administration of LPS (0.25 mg/kg/day). **(a)** Serum levels of alanine aminotransferase (ALT) in mice of each group (*n* = 5 per group). **(b**–**d)** mRNA level of tumour necrosis factor alpha (TNF-α), interleukin-6 (IL-6), and CD14 in mice of each group (*n* = 5 per group). **(e)** Western blotting for phosphorylated signal transducer and activator of transcription-3 (p-STAT3), total signal transducer and activator of transcription-3 (STAT3), and glyceraldehyde 3-phosphate dehydrogenase (GAPDH) in mice of the HFD+LPS-control group or HFD+LPS-RSV20-treated group. **(f)** Ratio of p-STAT3:STAT3 in mice of the HFD+LPS-control group or HFD+LPS-RSV20-treated group (*n* = 3–5 per group). Data represent relative expression compared with high-fat diet (HFD)+lipopolysaccharide (LPS)-control group mice after normalization with expression of β-actin mRNA and STAT3 protein. Error bars denote mean ± standard deviation. Significance was determined using the unpaired two-tailed Student’s *t*-test for parametric factors, and Mann–Whitney test for non-parametric factors. Asterisk denotes significant differences (*P < 0.05).

RSV significantly reduced serum levels of ALT as well as hepatic mRNA expression of TNF-α, IL-6, and CD14 in HFD fed-LPS-treated mice in a dose-dependent manner (Fig. 2a–d). Hepatic CD14 expression is upregulated by activation of the STAT3 pathway^17^. Hence, we measured alterations in levels of p-STAT3 as a marker of STAT3 activation in the liver. p-STAT3 levels were obviously and significantly inhibited by RSV treatment in HFD fed-LPS-treated mice (Fig. 2e,f). These results suggested that RSV may improve LPS-induced inflammation through inhibition of the STAT3-CD14 pathway in the liver.

### Target of RSV-mediated inhibition of STAT3 signalling in the liver is Kupffer cells

**Figure 3:**
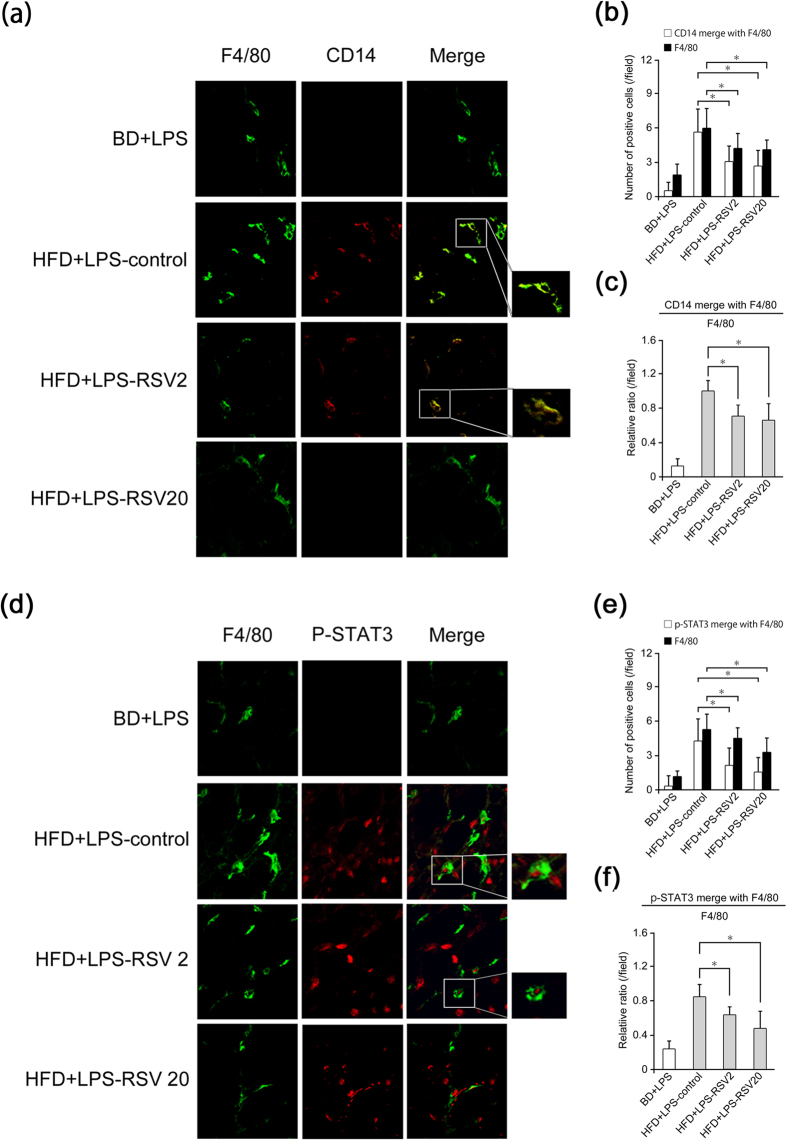
RSV relieved single low-dose LPS-induced hepatic inflammation through inhibition of STAT3 signalling and CD14 expression in Kupffer cells. (**a**–**f**) Mice were divided into four groups: mice receiving a basal diet (BD) in combination with lipopolysaccharide (LPS) administration (*n* = 5, BD+LPS group); mice receiving a high-fat diet (HFD) in combination with LPS administration (*n* = 5, HFD+LPS-control group); mice receiving a HFD mixed with resveratrol (RSV) 2 mg/kg/day (RSV2) in combination with LPS administration (*n* = 5, HFD+LPS-RSV2-treated group); mice receiving a HFD mixed with RSV 20 mg/kg/day (RSV20) in combination with LPS administration (*n* = 5, HFD+LPS-RSV20-treated group). NASH (inflammation) model in mice was created by feeding mice a HFD for 12 weeks in combination with administration of a single low-dose LPS (0.25 mg/kg/day) and RSV administered for the last 4 weeks. **(a)** Immunofluorescence of CD14 (red) with F4/80 (green). **(b)** Total number of CD14-positive cells and CD14-positive cells with F4/80 in mice of each group (*n* = 3–5 per group). **(c)** Relative ratio of total CD14-positive cells and CD14-positive cells with F4/80 in mice of each group (*n* = 3–5 per group). **(d)** Immunofluorescence of phosphorylated signal transducer and activator of transcription-3 (p-STAT3) (red) with F4/80 (green). **(e)** Total number of p-STAT3-positive cells and p-STAT3-positive cells with F4/80 in mice of each group (*n* = 3–5 per group). **(f)** Relative ratio of total p-STAT3-positive cells and p-STAT3-positive cells with F4/80 in mice of each group (*n* = 3–5 per group). Error bars denote mean ± standard deviation. Significance was determined using the unpaired two-tailed Student’s *t*-test. Asterisk denotes significant differences (*P < 0.05).

To investigate the target of RSV-mediated inhibition of STAT3 signalling in the liver, we undertook immunofluorescent staining with antibodies against CD14, p-STAT3 and F4/80. Previously, we reported that F4/80-positive cells represent Kupffer cells and CD14-positive cells represent activated Kupffer cells^17^. The number of F4/80-positive cells, CD14-positive cells, and the CD14:F4/80 ratio in the livers of HFD fed-LPS-treated mice were decreased significantly by RSV administration (RSV2 and RSV20) (Fig. 3a–c). The number of F4/80-positive cells, p-STAT3-positive cells, and the p-STAT3:F4/80 ratio in the livers of HFD fed-LPS-treated mice were decreased significantly by RSV administration (RSV2 and RSV20) (Fig. 3d–f). These results suggested that the target of RSV-mediated inhibition of STAT3 signalling in the inflamed livers of LPS-treated HFD-fed mice was activated Kupffer cells.

Additionally, to evaluate of the effects of RSV on liver inflammation in a single LPS-administered model of inflammation, we undertook immunohistochemistry with antibodies against F4/80 and neutrophil elastase (NE). Compared with LPS-treated HFD-fed mice, the number of both F4/80-positive cells and NE-positive cells in the livers of RSV-treated mice were decreased significantly by RSV administration (RSV2 and RSV20) (Supplementary Fig. S3a,b and Supplementary Fig. S4a,b). These results suggested that RSV may improve LPS-induced liver inflammation.

### Effect of RSV on NASH pathogenesis

**Figure 4:**
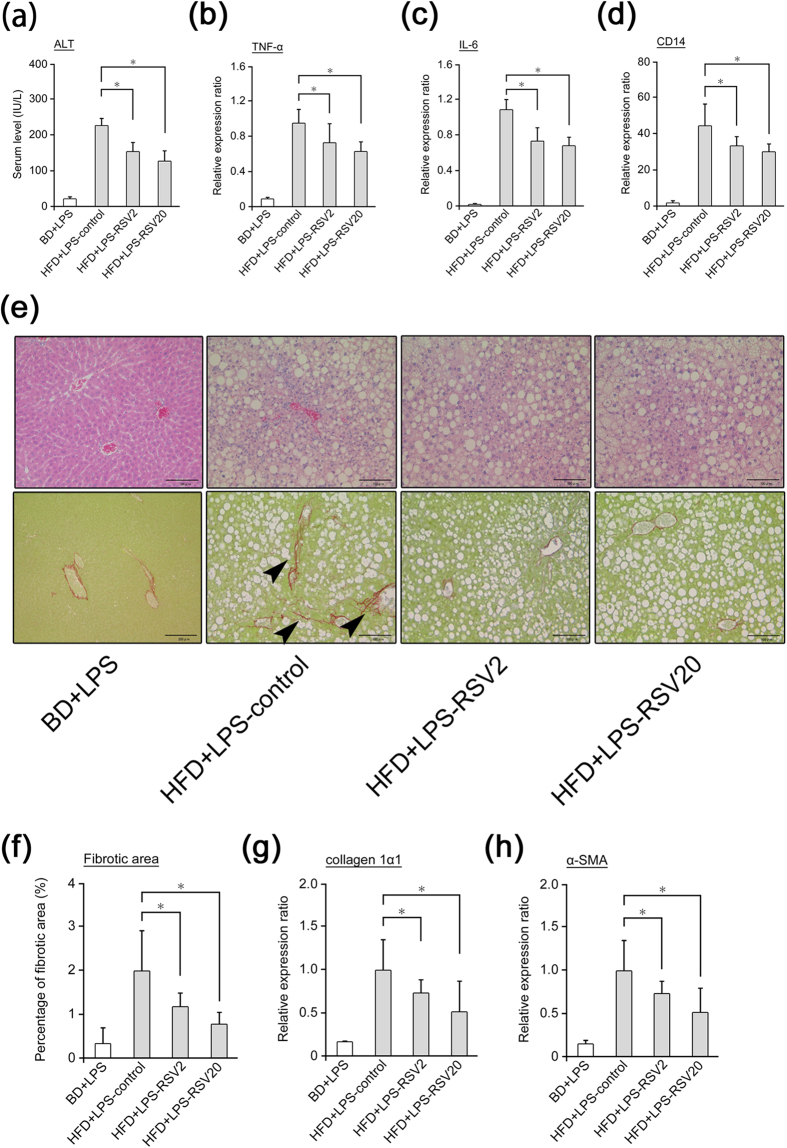
RSV can improve LPS-induced liver inflammation and fibrosis. (**a**–**h)** A mouse model of nonalcoholic steatohepatitis (NASH) was created by feeding mice a high-fat diet (HFD) for 12 weeks in combination with intraperitoneal injection of low-dose lipopolysaccharide (LPS) (0.25 mg/kg/day) for 4 weeks and RSV was administered for the last 4 weeks. Mice were divided into four groups: mice receiving a basal diet (BD) in combination with LPS administration (*n* = 5, BD+LPS group); mice receiving a high-fat diet (HFD) in combination with LPS administration (*n* = 5, HFD+LPS-control group); mice receiving a HFD mixed with resveratrol (RSV) 2 mg/kg/day (RSV2) in combination with LPS administration (*n* = 5, HFD+LPS-RSV2-treated group); mice receiving a HFD mixed with RSV 20 mg/kg/day (RSV20) in combination with LPS administration (*n* = 5, HFD+LPS-RSV20-treated group). **(a)** Serum levels of ALT in mice of each group (*n* = 5 per group), **(b**–**d)** mRNA level of tumour necrosis factor alpha (TNF-α), interleukin-6 (IL-6) and CD14 in mice of each group (*n* = 5 per group). **(e)** First row; haematoxylin and eosin staining (magnification: ×200, scale bar: 100 μm), second row; Sirius Red staining (magnification: ×200, scale bar: 100 μm), and fibrotic areas are stained red. Arrowheads denote extended fibrosis. **(f)** Fibrotic area in the liver (*n* = 5 per group). **(g**,**h)** mRNA level of collagen 1α1 and α-smooth muscle actin (α-SMA) in mice of each group (*n* = 5 per group). Error bars denote mean ± standard deviation. Significance was determined using the unpaired two-tailed Student’s *t*-test for parametric factors, and Mann–Whitney test for non-parametric factors. Asterisk denote significant differences (*P < 0.05).

To investigate the effect of RSV on NASH pathogenesis, a mouse model of NASH was used. The experimental design of the study is shown in Supplementary Fig. S1d. A mouse model of NASH was created by feeding mice a HFD for 12 weeks in combination with intraperitoneal injection of low-dose LPS (0.25 mg/kg/day) for 4 weeks in accordance with our previous study^17^. In humans, the NAFLD activity score (NAS) and fibrosis stage are used for the pathologic diagnosis of NASH^18^,^19^. We evaluated the NAS and fibrosis stage (Supplementary Table S3 and Supplementary Table S4) using haematoxylin and eosin (H&E) and Sirius red (SR) staining in liver tissues. Indeed, our NASH model was established in most HFD-fed LPS-control mice (Fig. 4e: first row and Supplementary Table. S5).

Mice were divided into four groups: (1) mice receiving a BD in combination with LPS administration (*n* = 5, BD+LPS group); (2) mice receiving a HFD in combination with LPS administration (*n* = 5, HFD+LPS-control group); (3) mice receiving a HFD mixed with RSV2 in combination with LPS administration (*n* = 5, HFD+LPS-RSV2-treated group); (4) mice receiving a HFD mixed with RSV20 in combination with LPS administration (*n* = 5, HFD+LPS-RSV20-treated group). Intraperitoneal injection of low-dose LPS itself did not elicit significant alterations in body weight, food intake, hepatic level of TG, or HOMA-IR among HFD+LPS-control-, HFD+LPS-RSV2-, or HFD+LPS-RSV20-treated groups (Table 2). However, significant decreases in levels of ALT, inflammatory cytokines (TNF-α, IL-6), CD14 and HOMA-IR were observed in RSV-treated groups (Fig. 4a–d, Table 2). In addition, as shown in Fig. 4e and supplementary Table S5, we undertook histological evaluation of the liver upon RSV treatment using NAS and fibrosis stage. The increased NAS and fibrosis stage in HFD-fed LPS-administrated mice reduced significantly in RSV-treated mice. In particular, lobular inflammation and hepatocyte ballooning in NAS decreased considerably (Fig. 4e and Supplementary Table. S5). Development of hepatic fibrosis induced by low-dose LPS was suppressed dramatically by RSV treatment according to SR and Masson Trichrome staining (Fig. 4e; second row, Fig. 4f and Supplementary Fig S5a,b). Suppression of the development of hepatic fibrosis by RSV treatment was confirmed by expression of the mRNA of collagen 1α1 and α-SMA in the liver (Fig. 4g,h). These results demonstrated that RSV treatment prevented fibrosis and inflammation in our NASH model of mice.

### RSV inhibits leptin-induced phosphorylation of STAT3 in RAW264.7 cells

Previously, we reported that obesity-induced increases in leptin release cause activation of the STAT3 pathway and result in hepatic expression of CD14^17^. Increased hepatic expression of CD14 can lead to hyper-responsivity against low-dose LPS, resulting in excessive inflammation and fibrosis (i.e., NASH)^17^. Therefore, we investigated the effect of RSV on leptin-mediated activation of STAT3 *in vitro* using a cultured murine monocyte/macrophage cell line: RAW264.7.

**Figure 5:**
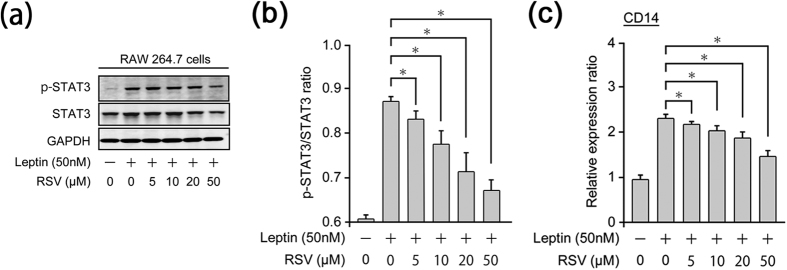
RSV inhibits leptin-induced expression of p-STAT3 in RAW264.7 cells. (**a)** Leptin alone or leptin with resveratrol (RSV)-induced protein levels of phosphorylated signal transducer and activator of transcription-3 (p-STAT3) in RAW 264.7 cells (murine monocyte/macrophage cell line) using immunoblotting analyses. **(b)** Leptin alone or leptin with RSV-induced ratio of p-STAT3:STAT3 in RAW 264.7 cells (*n* = 5). **(c)** Leptin alone or leptin with RSV-induced CD14 mRNA expression in RAW 264.7 cells (*n* = 4). Error bars denote mean ± standard deviation. Significance was determined using ANOVA with Scheffe’s multiple testing correction. Asterisk indicates significant differences (*P < 0.05).

Application of recombinant leptin into the culture medium increased protein levels of p-STAT3 and CD14 mRNA dramatically in RAW264.7 cells (Fig. 5a–c). RSV inhibited protein levels of p-STAT3 and CD14 mRNA in a dose-dependent manner. Significant inhibition of the p-STAT3:STAT ratio (a marker of STAT3 activation) and expression of CD14 mRNA was observed at RSV concentrations >5 μM, which is a very high concentration compared with systemic RSV concentrations documented in clinical studies (C_max_ < 4 μM, C_mean_ ≪ 1 μM)^20^,^21^,^22^.

## Discussion

In the present study, we clearly demonstrated that RSV inhibited fibrosis and inflammation (but not steatosis) dramatically in a mouse model of NASH. This is the first report on the protective effect of RSV on NASH progression.

Several studies in models of obesity in rodents have demonstrated that RSV reduces fat accumulation and weight^23^,^24^,^25^. In contrast to our study, Nishikawa *et al*. reported that RSV improved liver steatosis in mice using the same model of a HFD in mice^26^. However, the administration period and dose of RSV between our study and their study were different.

Recent clinical trials have shown that RSV improves steatosis in NAFLD pathogenesis^27^,^28^. However, recent studies in NAFLD patients have shown that RSV does not stop NAFLD development^29^. Differences among these studies include the administration period, dose, and selection bias. Thus, the effect of RSV on steatosis in NAFLD patients is controversial. We hypothesized that the effect of RSV on steatosis may be limited: the present study supports this hypothesis.

Recently, it has been shown that circulating levels of LPS are increased in NAFLD patients^30^, and that LPS can induce release of pro-inflammatory (and potentially fatigue-inducing) cytokines such as TNF-α and IL-6^31^,^32^. Previously, we demonstrated that leptin induces CD14 expression *via* activation of STAT3 signalling in Kupffer cells, resulting in enhanced responsivity against low-dose LPS in the liver^17^. Also, CD14 is an important regulatory factor in expression of the inflammatory response to LPS, and enhances the effect of LPS in Kupffer cells considerably^33^,^34^,^35^,^36^,^37^. Clinical studies have shown that CD14 polymorphism is a risk factor for NASH pathogenesis^38^. Therefore, CD14 expression is closely related to NASH pathogenesis. In addition, CD14 is a co-receptor that is detected in two forms: a glycosylphosphatidylinositol-anchored membrane protein (mCD14) and a soluble serum protein (sCD14) lacking the anchor protein^37^. Several reports have shown that sCD14 is shed from the surface of mCD14-expressing cells^39^,^40^. sCD14 has been reported to enhance endotoxin clearance from serum in NASH with liver fibrosis^41^. Previously, we showed that hepatic expression of CD14 (mCD14) mRNA is much higher in NASH patients than in NAFL subjects^17^. Moreover, those results demonstrated that hepatic expression of CD14 (mCD14) may be an important factor in NASH development because it enhances the hepatic inflammatory response against LPS derived from gut bacteria. Therefore, inhibition of CD14 (mCD14) expression in the liver is important for the prevention of fibrosis and inflammation with respect to NASH phenotypes. Therefore, we investigated the anti-inflammatory and anti-fibrotic effects of RSV *via* inhibition of CD14 expression in a mouse model of NASH.

Previously, we reported that F4/80-positive cells represent Kupffer cells and CD14-positive cells represent activated Kupffer cells^17^. Our present data clearly indicate that CD14-positive cells are identified as Kupffer cells but not other cells. Activation of Kupffer cells accompanied by CD14 expression along with cytokine release was suppressed dramatically by RSV administration *via* inhibition of the STAT3 pathway in a mouse model of NASH.

Also, inflammatory cells in the liver exist mainly as Kupffer cells and neutrophils. In particular, in the endotoxin model, recruitment of neutrophils into the liver results in hepatocellular injury^42^. Therefore, we investigated the effect of RSV on not only Kupffer cells but also neutrophils in our model. We found that RSV reduced LPS-induced increases in Kupffer cells as well as neutrophils in the liver.

Inhibition of the STAT3 pathway by RSV administration was confirmed *in vitro* using cultured cells. Bhardwaj *et al*. reported that RSV inhibits activation of the STAT3 pathway^43^. Therefore, our observations support the work of Bhardwaj *et al*. RSV concentrations used in our *in vitro* study were much higher than those of clinical studies^20^,^21^,^22^. However, the suppressive effect of 5 μM RSV on STAT3 activation and CD14 expression appeared very weak (even though significant suppression by 5 μM RSV was observed). The reason for the discrepancy between our data and clinical studies may have been due to different: (i) RSV concentrations in liver tissue and blood; (ii) drug sensitivities and pharmacokinetics between rodents and humans. Also, other mechanisms might have been involved in RSV-mediated inhibition of inflammation and fibrosis. Further investigations are required to clarify the mechanism of action of the protective effect of RSV on NASH.

**Figure 6:**
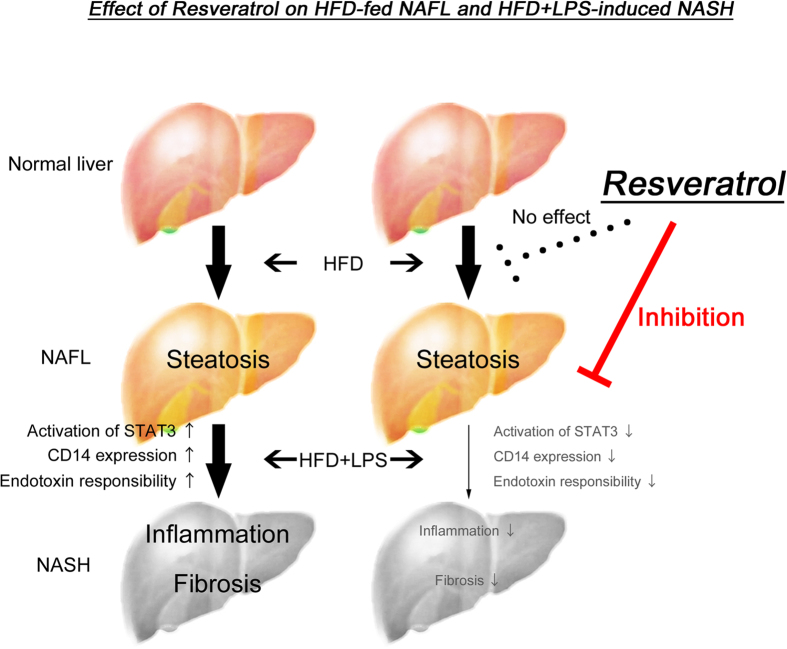
Effect of resveratrol on NAFL or LPS-induced NASH. Resveratrol improved inflammation, and prevented liver fibrosis in nonalcoholic steatohepatitis (NASH) *via* inhibition of lipopolysaccharide (LPS) hyper-responsivity but not steatosis in nonalcoholic fatty liver (NAFL) by feeding of a high-fat diet.

Our data led to a hypothesis on the effect of RSV on NASH inhibition: a HFD causes increases in leptin release from adipose tissue that result in CD14 expression in Kupffer cells, which leads to hyper-responsivity against low-dose LPS. Activated Kupffer cells/inflammatory cells in the liver cause inflammation and fibrosis accompanied by increases in the release of various cytokines/chemokines, resulting in NASH. RSV administration can inhibit activation of the STAT3 pathway, thereby resulting in suppression of CD14 expression in Kupffer cells in the liver. Inhibition of CD14 expression is critical for the progression from steatosis to steatohepatitis. Therefore, the inhibitory effect of RSV may be an important mechanism in the prevention of NASH progression (Fig. 6). Further investigations are required to clarify the other mechanisms of action of RSV.

In summary, we showed that, in a mouse model of NASH/NAFL, RSV administration can improve inflammation and fibrosis, but not steatosis, *via* inhibition of LPS reactivity that is due to CD14 expression in Kupffer cells. Our results suggest that RSV could be a candidate agent for the NASH treatment.

## Methods

### Reagents, substances and antibodies

Lipopolysaccharide (LPS; derived from *Escherichia coli*) was purchased from Sigma–Aldrich (0.25 mg/kg; Saint Louis, MO, USA). Resveratrol (RSV, >99% *trans*-resveratrol) was obtained from Fluxomo (Stenlose, Denmark). Murine recombinant leptin was purchased from PeproTech (Rocky Hill, NJ, USA). Primary antibodies (signal transducer and activator of transcription (STAT)3, phosphorylated-signal transducer and activator of transcription (p-STAT)3, CD14 were obtained from Cell Signaling Technology (Danvers, MA, USA), F4/80 was obtained from eBioscience. Horseradish peroxidase-conjugated secondary antibody was purchased from Cell Signaling Technology. All other chemicals and substances were of reagent grade.

### Animal experiments

Eight-week-old male C57BL/6J mice were purchased from Japan CLEA (Tokyo, Japan). Mice were housed in a room with a light–dark cycle of 12 h–12 h maintained at 22–24 °C, and fed rodent chow and water *ad libitum*. The basal diet (BD) contained 22% protein, 6% fat, and 47% carbohydrate. The high-fat diet (HFD) contained 20% protein, 60% fat, and 20% carbohydrate^17^.

Animal experiments were carried out according to the illustrations shown in Supplementary Fig. S1. The NAFL (steatosis) model, which involved random division of mice into four groups (*n* = 5), was created by feeding mice a HFD for 12 weeks in accordance with our previous studies^17^,^26^,^44^ (Supplementary Fig. S1a,b). Two concentrations of RSV (2 mg/g and 20 mg/g) were mixed with a HFD and administered for the last 2 weeks (Supplementary Fig. S1a) or 4 weeks (Supplementary Fig. S1b). Estimated dose was calculated according to the following formula:


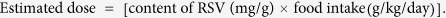


Hence, the content of 0.763 mg/g RSV was calculated to be 2.013 mg/kg/day, and that of 7.76 mg/kg RSV was 20.20 mg/kg/day. We described the doses of RSV as 2 mg/kg/day (RSV2) and 20 mg/kg/day (RSV20). The NASH (inflammation) model, which involved random division of mice into four groups (*n* = 5), was created by feeding mice a HFD for 12 weeks in combination with administration of a single low-dose LPS (0.25 mg/kg/day) in accordance with our previous study^17^ (Supplementary Fig. S1c).

The NASH (inflammation and fibrosis) model, which involved division of mice into four groups (*n* = 5), was created by feeding mice a HFD for 12 weeks in combination with administration of a low-dose of LPS (0.25 mg/kg/day) for 4 weeks in accordance with our previous study^11^ (Supplementary Fig. S1d). In this model, marked elevation of levels of alanine aminotransferase (ALT) accompanied by obvious fibrosis and inflammation, which are typical phenotypes of NASH, was observed at 12 weeks^17^. RSV2 and RSV20 were administered to mice for the last 4 weeks. At 12 weeks, mice were killed and samples collected for each type of analysis. All animal experiments were performed in accordance with the guidelines of the Ethics Committee of the Medical school of Yokohama City University and were approved by the Animal Care and Use Committee of Yokohama City University.

### Cell culture

For *in vitro* experiments, the murine monocyte/macrophage-cell line RAW264.7 was obtained from American Type Culture Collection (Manassas, VA, USA). Cells were cultured at 37 °C in an atmosphere of 5% CO_2_ in Dulbecco’s modified Eagle’s medium supplemented with 100 units/ml penicillin and 100 mg/ml streptomycin plus 10% fetal bovine. Murine recombinant leptin (50 nM) was added to the culture medium to induce activation of the STAT3 pathway^17^,^45^. Application of RSV (0, 5, 10, 20 and 50 μM) to the culture medium was undertaken. After 6 h of incubation, cells were harvested and samples prepared.

### Biochemical analyses

Serum levels of ALT, glucose and insulin were measured by a local laboratory (SRL, Tokyo, Japan). The extent of insulin resistance was calculated using the homeostasis model for the assessment of insulin resistance (HOMA-IR) according to the following formula^46^:


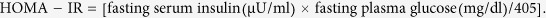


### Liver histology

Liver samples were excised and embedded in Tissue-Tek^®^ OCT compound (Sakura FineTek USA, Torrance, CA, USA) or paraffin for histological analyses. Formalin-fixed and paraffin-embedded sections were processed routinely with haematoxylin and eosin (H&E) and Sirius red (SR) staining. To evaluate fat deposition, OCT-embedded samples were stained with Oil Red O. To quantify the fibrotic area of SR staining, images of five random fields of each section were processed with Photoshop Elements v13 (Adobe Systems, San Jose, CA, USA). Each value was expressed as the percentage of the total area of the section. NAS and fibrosis stage were scored by an experienced pathologist (NY) blinded to the experimental procedures according to the criteria set out by Brunt *et al*.^18^,^19^ (Supplementary Table S3 and Supplementary Table S4).

### Immunofluorescence and immunoblotting

Immunofluorescence was carried out on 7-mm cryostat liver sections. Sections were incubated with primary antibodies, and stained with Alexa Fluoro-conjugated secondary antibodies (Cell Signaling Technology). Proteins were incubated with primary antibodies, and horseradish-conjugated secondary antibody (Cell Signaling Technology). Primary antibodies were p-STAT3, STAT3 and CD14 (Cell Signaling Technology).

### RNA isolation and real-time polymerase chain reaction (PCR) analyses

Total RNA was isolated from samples using an RNeasy Mini kit (Qiagen, Stanford, VA, USA). Reverse transcription (RT) to produce cDNA was undertaken using a TaqMan^®^ Gold RT-PCR kit (Applied Biosystems, Foster City, CA, USA) according to manufacturer instructions. Reaction mixtures (100 μL) contained 2.5 μg of total RNA and the reaction was carried out for 50 min at 48 °C; then, reverse transcriptase was inactivated by heating samples to 95 °C for 5 min. mRNA expression of CD14, tumour necrosis factor (TNF)-α, interleukin (IL)-6, α-smooth muscle actin (α-SMA), collagen 1α1 and β-actin in liver tissue was determined using fluorescence-based RT-PCR and an ABI PRISM 7700 Sequence Detection System (Life Technologies, Carlsbad, CA, USA). RT-PCR was carried out using a TaqMan Universal PCR Master Mix Reagent. Values in all samples were normalised to expression of the endogenous control. Levels of CD14, TNF-α, IL-6, collagen 1α1, α-SMA, and β-actin were determined by quantitative real-time RT-PCR.

### Determination of hepatic levels of triglyceride (TG)

Lipids were extracted from liver tissue (50 mg) using chloroform:methanol (2:1) as described by Folch *et al*. TG levels in serum and the liver were measured by the GPO/HMMPS method with L-Type Wako TG (Wako Pure Chemical Industries, Tokyo, Japan)^47^.

### Statistical analyses

Statistical analyses were carried out using SPSS v12 (IBM. Armonk, NY, USA). Data are the mean ± SD. Differences between the two groups were assessed using the unpaired two-tailed Student’s *t*-test for parametric factors, and Mann–Whitney test for non-parametric factors. Datasets involving more than two groups were assessed by ANOVA with Scheffe’s multiple testing correction. *P* < 0.05 was considered significant.

## Additional Information

**How to cite this article**: Kessoku, T. *et al*. Resveratrol ameliorates fibrosis and inflammation in a mouse model of nonalcoholic steatohepatitis. *Sci. Rep*. **6**, 22251; doi: 10.1038/srep22251 (2016).

## Supplementary Material

Supplementary Information

## Figures and Tables

**Table 1:** Characteristics of the NAFL model group and 4-week RSV-treated group.

Variable	BD group (*n* = 5)	HFD-control group (*n* = 5)	HFD-RSV2- treated group (*n* = 5)	HFD-RSV20- treated group (*n* = 5)
Initial body weight (g)	21.8 ± 0.41	20.1 ± 0.26	22.9 ± 0.18 (n.s.)	20.7 ± 0.25 (n.s.)
Final body weight (g)	31.5 ± 0.84	43.5 ± 3.59	42.7 ± 3.10 (n.s.)	42.0 ± 3.81 (n.s.)
Water intake (ml/day/mouse)	6.65 ± 0.57	8.54 ± 0.69	8.14 ± 0.90 (n.s.)	8.34 ± 0.85 (n.s.)
Food intake (g/day/mouse)	2.54 ± 0.69	2.74 ± 0.64	2.51 ± 0.87 (n.s.)	2.69 ± 0.61 (n.s.)
Liver weight (g)	1.12 ± 0.16	2.48 ± 0.46	2.49 ± 0.63 (n.s.)	2.32 ± 0.49 (n.s.)
White-fat weight (g)	0.71 ± 0.20	1.61 ± 0.56	1.55 ± 0.61 (n.s.)	1.75 ± 0.55 (n.s.)
HOMA-IR	1.8 ± 0.6	3.55 ± 0.81	3.37 ± 0.69 (n.s.)	3.30 ± 0.55 (n.s.)

BD: basal diet, HFD: high-fat diet, RSV: resveratrol, RSV2: resveratrol 2 mg/kg/day, RSV20: resveratrol 20 mg/kg/day, HOMA-IR: homeostasis model for the assessment of insulin resistance. Data are the mean ± standard deviation; n.s., no significant difference *vs*. the HFD-control group. (*n* = 5 per group).

**Table 2:** Characteristics of the LPS-induced NASH model group and RSV-treated group.

Variable	BD+LPS group (*n* = 5).	HFD+LPS- control group (*n* = 5).	HFD+LPS- RSV2- treated group (*n* = 5).	HFD+LPS- RSV20- treated group (*n* = 5).
Initial body weight (g)	23.3 ± 0.28	22.1 ± 0.21	23.3 ± 0.28 (n.s.)	23.0 ± 0.28 (n.s.)
Final body weight (g)	28.3 ± 1.65	39.9 ± 1.12	38.2 ± 1.65 (n.s.)	39.6 ± 1.91 (n.s.)
Water intake (ml/d/mouse)	7.87 ± 0.28	8.12 ± 0.53	8.08 ± 0.30 (n.s.)	8.11 ± 0.49 (n.s.)
Food intake (g/d/mouse)	2.35 ± 0.65	2.39 ± 0.71	2.49 ± 0.82 (n.s.)	2.39 ± 0.95 (n.s.)
Liver weight (g)	1.13 ± 0.21	2.70 ± 0.55	2.48 ± 0.53 (n.s.)	2.40 ± 0.61 (n.s.)
White-fat weight (g)	0.63 ± 0.23	1.79 ± 0.46	1.82 ± 0.61 (n.s.)	1.77 ± 0.52 (n.s.)
HOMA-R	2.12 ± 0.41	3.95 ± 0.50	3.29 ± 0.71^#^	2.85 ± 0.75^#^

BD: basal diet, HFD: high-fat diet, LPS: lipopolysaccharide, RSV: resveratrol, RSV2: resveratrol 2 mg/kg/day, RSV20: resveratrol 20 mg/kg/day, HOMA-IR: homeostasis model for the assessment of insulin resistance. Data are the mean ± standard deviation, ^#^*P* < 0.05, significant difference *vs*. HFD+LPS-control group. (*n* = 5 per group).
