# Supplementary material for: Resveratrol ameliorates fibrosis and inflammation in a mouse model of nonalcoholic steatohepatitis
Source: Sci Rep. 2016 Feb 25;6:22251. doi: 10.1038/srep22251 (PMC4766502; doi:10.1038/srep22251)
Supplement: Supplementary Information [file srep22251-s1.doc]

**Supplementary information**

**Resveratrol ameliorates fibrosis and inflammation in a mouse model of nonalcoholic steatohepatitis**

Takaomi Kessoku^1^, Kento Imajo^1^, Yasushi Honda^1^, Takayuki Kato^1^, Yuji Ogawa^1^, Wataru Tomeno^1^, Shingo Kato^1^, Hironori Mawatari^1^, Koji Fujita^1^, Masato Yoneda^1^, Yoji Nagashima^2^, Satoru Saito^1^, Koichiro Wada^3^, Atsushi Nakajima^1^*

^1^Department of Gastroenterology and Hepatology, Yokohama City University Graduate School of Medicine, 3-9 Fukuura, Kanazawa-ku, Yokohama, 236-0004, Japan

^2^Department of Department of Surgical Pathology, Tokyo Women`s Medical University, 8-1, Kawada-cho, Shinjuku-ku, Tokyo 162-8666, Japan.

^3^Department of Pharmacology, Shimane University Faculty of Medicine,

89-1 Enya-cho, Izumo, Shimane, 693-8501, Japan

**Address correspondence to:** Department of Gastroenterology and Hepatology, Yokohama City University Graduate School of Medicine, 3-9 Fukuura, Kanazawa-ku, Yokohama 236-0004, Japan, Atsushi Nakajima; Telephone: +81-45-787-2640; Fax: +81-45-784-3546; E-mail: [nakajima-tky@umin.ac.jp](mailto:nakajima-tky@umin.ac.jp)

**Supplementary Figure S1. Experimental design of the study.**


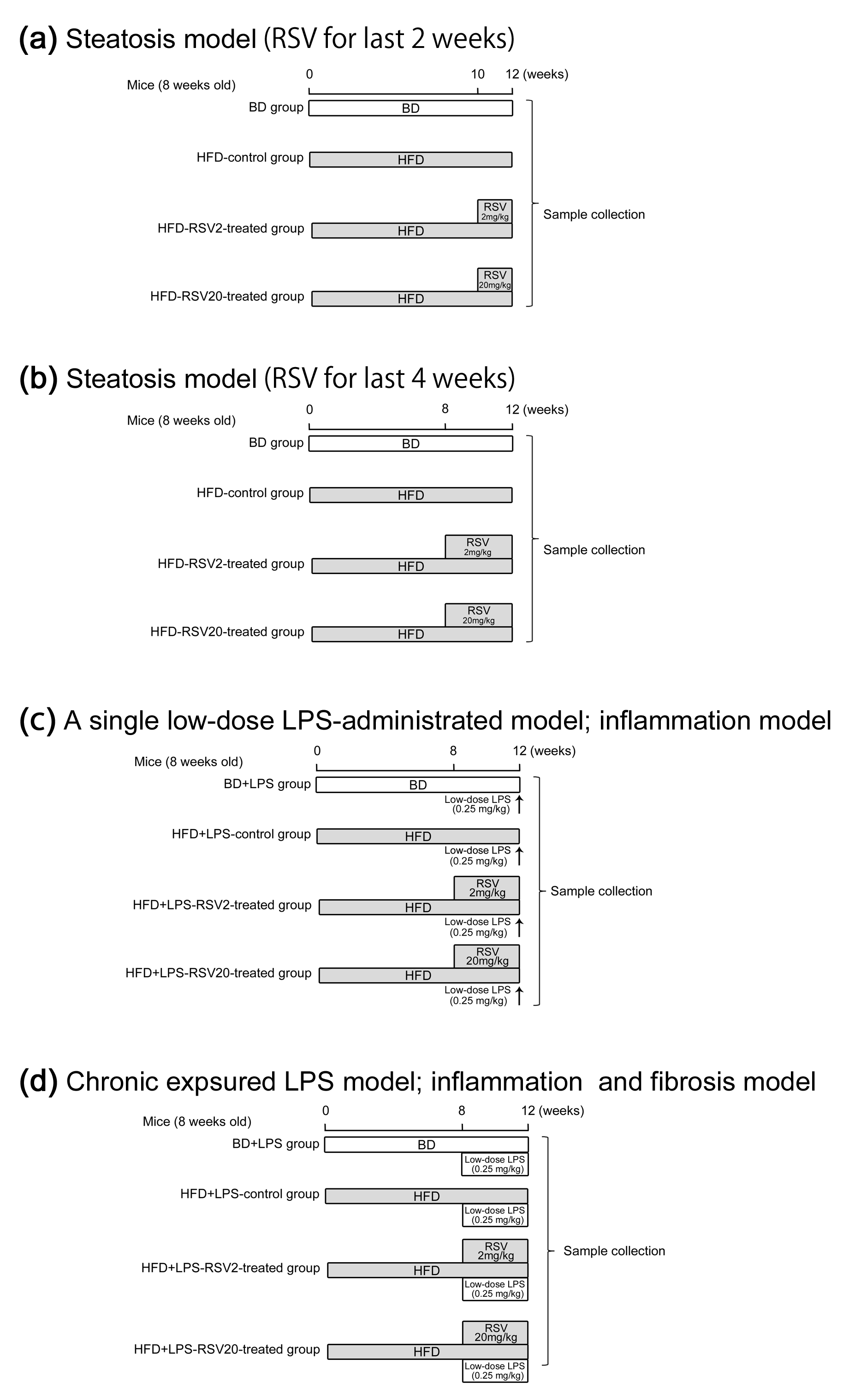


Mice were divided into four groups. Mice of each group fed a basal diet (BD), high-fat diet (HFD), HFD+resveratrol (RSV) 2 mg/kg/day (RSV2), or HFD+RSV 20 mg/kg/day (RSV20). **(a)** Effects of RSV on a nonalcoholic fatty liver (NAFL) (steatosis) mouse model. RSV administered for the last 2 weeks (*n*=5 per group). **(b)** Effects of RSV on a NAFL (steatosis) mouse model. RSV administered for the last 4 weeks (*n*=5 per group). **(c)** Effects of RSV on a single-dose lipopolysaccharide (LPS) (0.25 mg/kg)-administered nonalcoholic steatohepatitis (NASH) (inflammation) model (*n*=5 per group). **(d)** Effects of RSV on continuous LPS (0.25 mg/kg)-administrated NASH (inflammation and fibrosis) mouse model. (*n*=5 per group).

**Supplementary Figure S2. 2-week administration of resveratrol (RSV) does not reduce hepatic fat accumulation but RSV can inhibit mRNA levels of hepatic CD14.**


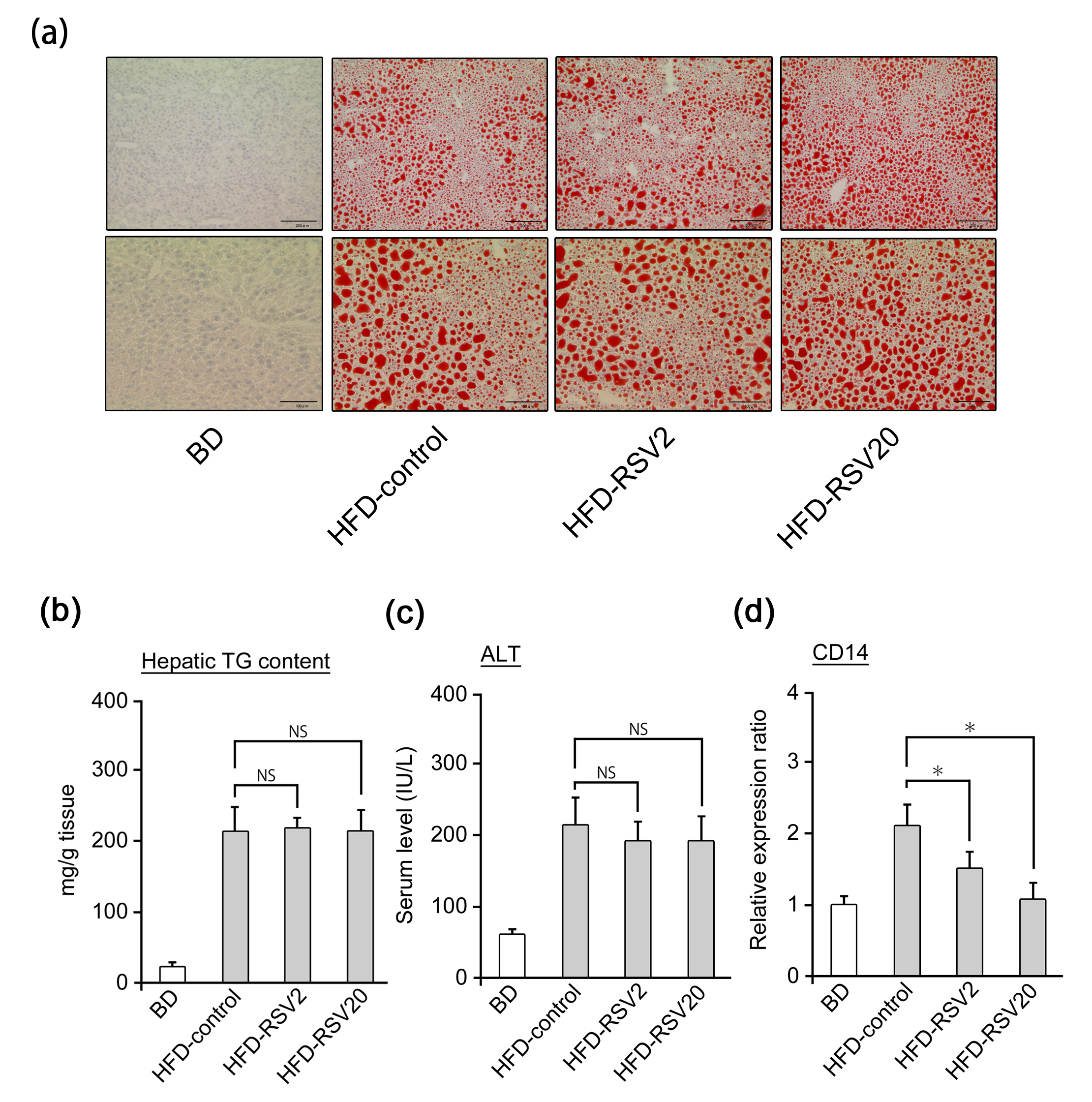


**(a)** After the particular time period of the experiment, liver tissue was isolated from mice, and liver sections stained with Oil Red O. Histological photomicrograph of Oil Red O staining (first row: magnification ×100, scale bar: 200 µm; second row: magnification ×200, scale bar: 100 µm). Lipid droplets are stained red. **(b)** Measurement of accumulation of triglyceride (TG) in the livers of mice. After the particular time period of the experiment, TG in whole liver was measured as described in the Methods section. (*n*=5 per group). **(c)** Serum levels of alanine aminotransferase (ALT) (*n*=5 per group). **(d)** Expression of CD14 mRNA in the liver quantified using real-time RT-PCR. (*n*=5 per group). Data represent relative expression compared with high-fat diet (HFD)-control group mice after normalization with expression of β-actin mRNA. BD: basal diet, RSV: resveratrol, RSV2: resveratrol 2 mg/kg/day, RSV20: resveratrol 20 mg/kg/day. Error bars denote mean ± standard deviation. Significance was determined using the unpaired two-tailed Student’s *t*-test for parametric factors, and Mann–Whitney test for non-parametric factors. Asterisk indicates significant differences (**P*<0.05). n.s.: not significant.

**Supplementary Figure S3. RSV relieved single low-dose LPS-induced Kupffer cells.**


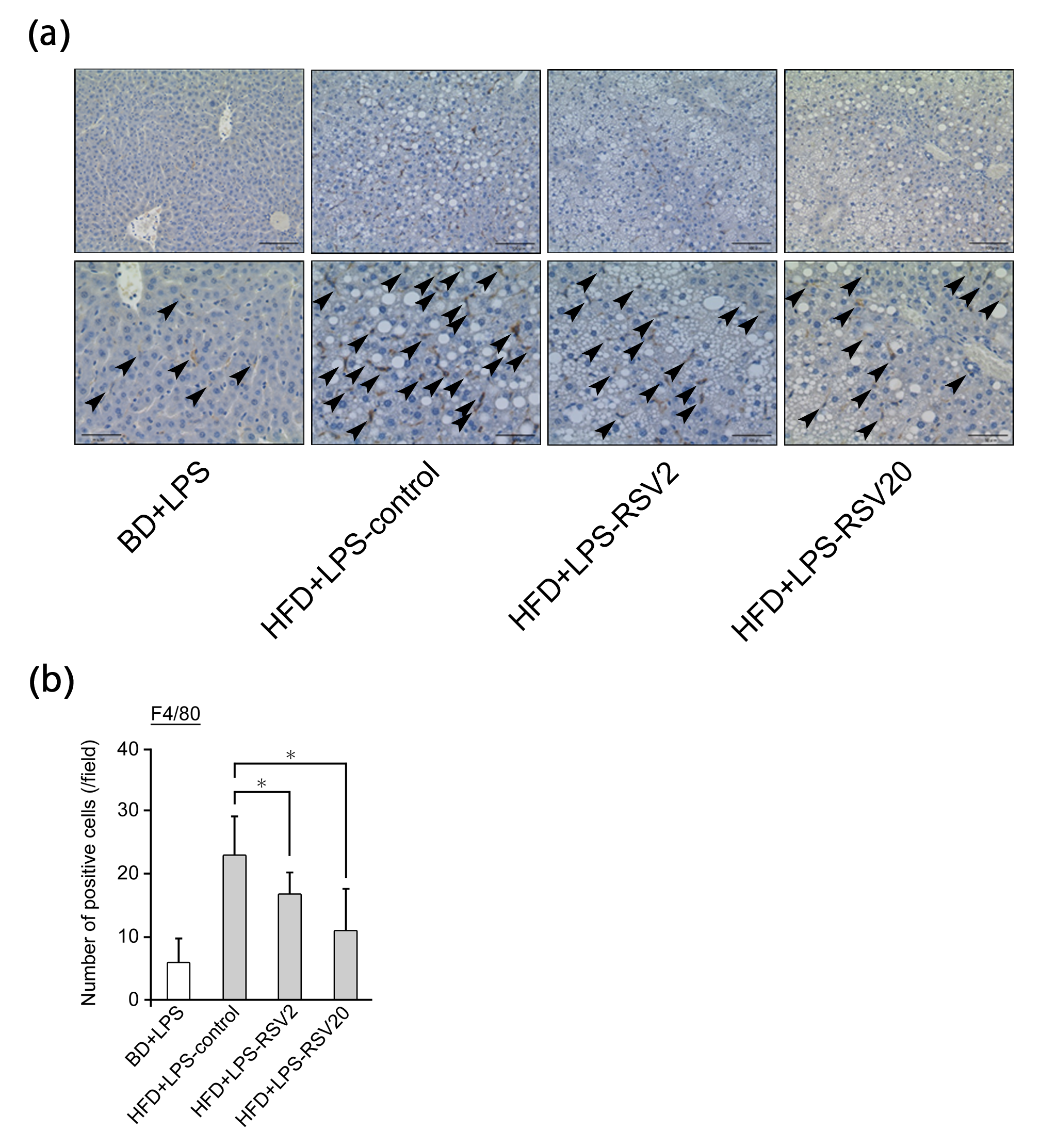


**(a-b)** Mice were divided into four groups: mice receiving a basal diet (BD) in combination with lipopolysaccharide (LPS) administration (*n*=5, BD+LPS group); mice receiving a high-fat diet (HFD) in combination with LPS administration (*n*=5, HFD+LPS-control group); mice receiving a HFD mixed with resveratrol (RSV) 2 mg/kg/day (RSV2) in combination with LPS administration (*n*=5, HFD+LPS-RSV2-treated group); mice receiving a HFD mixed with RSV 20 mg/kg/day (RSV20) in combination with LPS administration (*n*=5, HFD+LPS-RSV20-treated group). A NASH (inflammation) model in mice was created by feeding mice a HFD for 12 weeks in combination with administration of a single low-dose LPS (0.25 mg/kg/day) and RSV administered for the final 4 weeks.

**(a)** Immunohistochemistry of F4/80-stained liver sections. First row (magnification: ×200, scale bar: 100 µm), second row (magnification: ×400, scale bar: 50 µm). Arrowheads denote F4/80-positive cells. **(b)** Total number of F4/80-positive cells in mice of each group (*n*=3-5 per group). Error bars denote mean ± standard deviation. Significance was determined using the Mann–Whitney test for non-parametric factors. Asterisk denotes significant differences (**P*<0.05).

**Supplementary Figure S4. RSV relieved single low-dose LPS-induced neutrophil recruitment.**


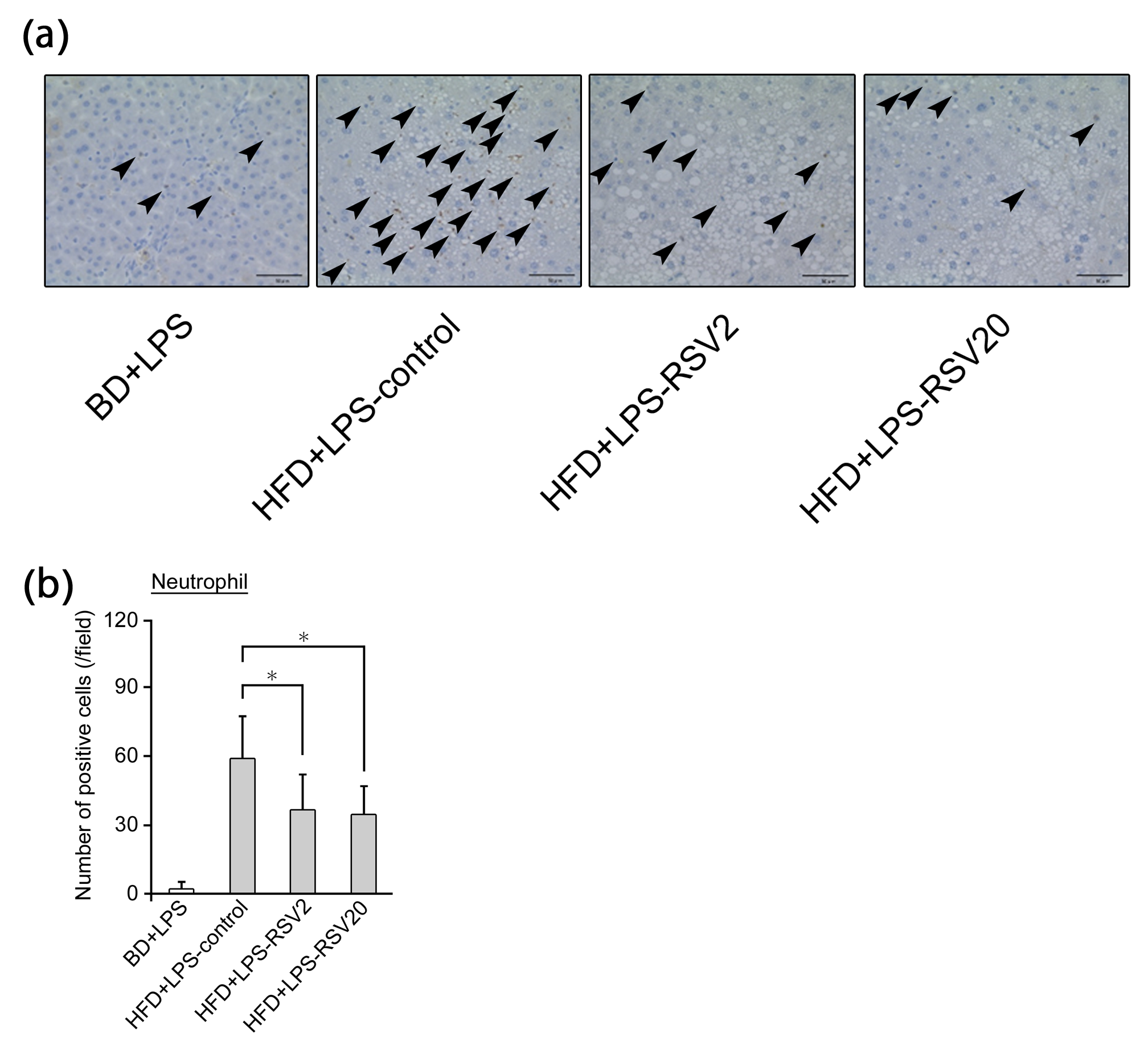


**(a-b)** Mice were divided into four groups: mice receiving a basal diet (BD) in combination with lipopolysaccharide (LPS) administration (*n*=5, BD+LPS group); mice receiving a high-fat diet (HFD) in combination with LPS administration (*n*=5, HFD+LPS-control group); mice receiving a HFD mixed with resveratrol (RSV) 2 mg/kg/day (RSV2) in combination with LPS administration (*n*=5, HFD+LPS-RSV2-treated group); mice receiving a HFD mixed with RSV 20 mg/kg/day (RSV20) in combination with LPS administration (*n*=5, HFD+LPS-RSV20-treated group). A NASH (inflammation) model in mice was created by feeding mice a HFD for 12 weeks in combination with administration of a single low-dose LPS (0.25 mg/kg/day) and RSV administered for the final 4 weeks.

**(a)** Immunohistochemistry of neutrophil elastase (NE) -stained liver sections (magnification: ×200, scale bar: 100 µm). Arrowheads denote NE-positive cells. **(b)** Total number of NE-positive cells in mice of each group (*n*=3-5 per group). Error bars denote mean ± standard deviation. Significance was determined using the Mann–Whitney test for non-parametric factors. Asterisk denotes significant differences (**P*<0.05).

**Supplementary Figure S5. RSV can improve liver fibrosis evaluated by Masson Trichrome staining in LPS-induced NASH model of mice.**
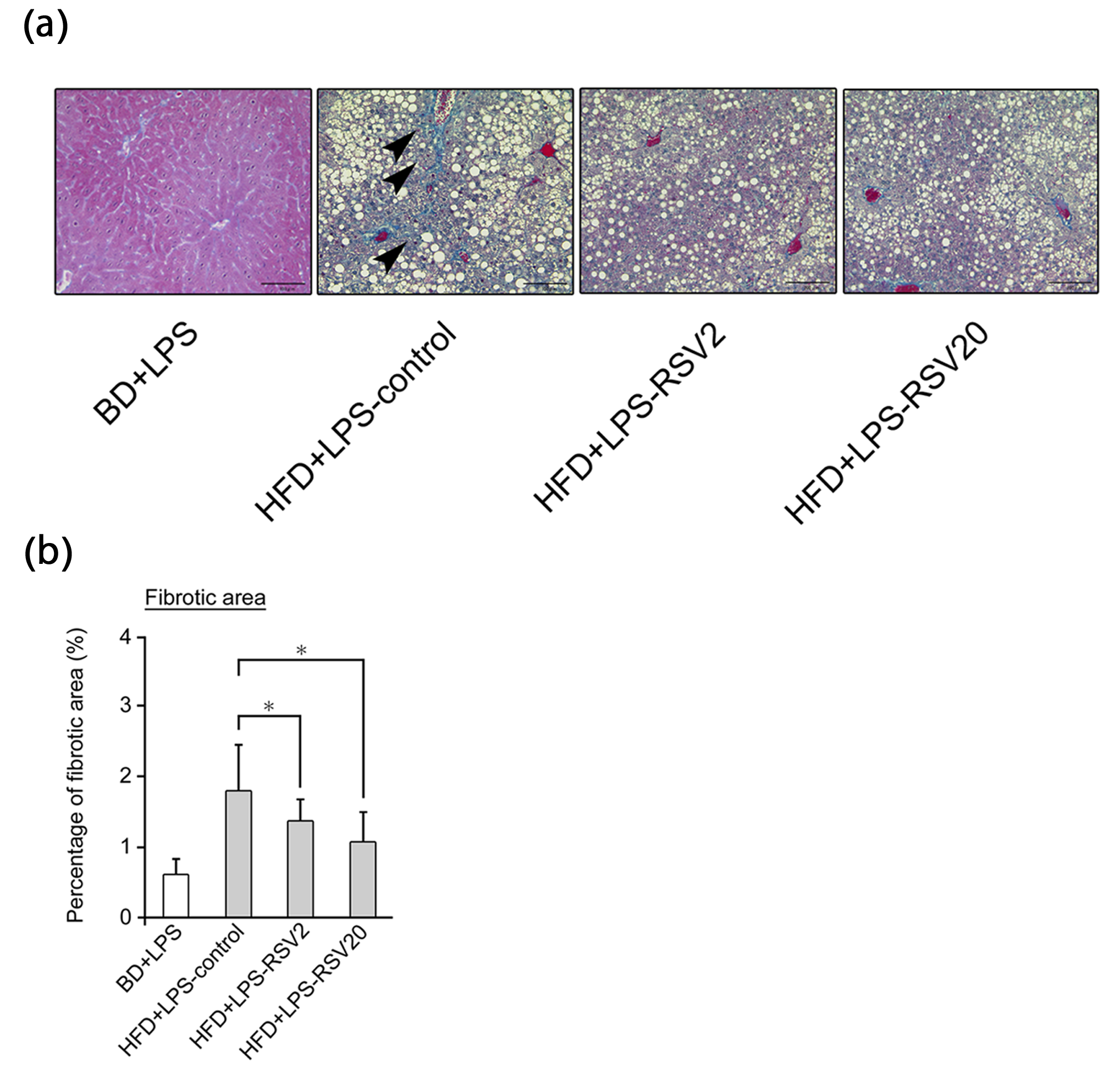


**(a-b)** A mouse model of nonalcoholic steatohepatitis (NASH) was created by feeding mice a high-fat diet (HFD) for 12 weeks in combination with intraperitoneal injection of low-dose lipopolysaccharide (LPS) (0.25 mg/kg/day) for 4 weeks and RSV was administered for the last 4 weeks. Mice were divided into four groups: mice receiving a basal diet (BD) in combination with LPS administration (*n*=5, BD+LPS group); mice receiving a high-fat diet (HFD) in combination with LPS administration (*n*=5, HFD+LPS-control group); mice receiving a HFD mixed with resveratrol (RSV) 2 mg/kg/day (RSV2) in combination with LPS administration (*n*=5, HFD+LPS-RSV2-treated group); mice receiving a HFD mixed with RSV 20 mg/kg/day (RSV20) in combination with LPS administration (*n*=5, HFD+LPS-RSV20-treated group). **(a)** Masson Trichrome staining (magnification: ×200, scale bar: 100 µm), and fibrotic areas are stained blue. Arrowheads denote extended fibrosis. **(b)** Fibrotic area in the liver (*n*=5 per group). Error bars denote mean ± standard deviation. Significance was determined using the Mann–Whitney test for non-parametric factors. Asterisk indicates significant differences (**P*<0.05).

**Supplementary Table S1. Characteristics of NAFL model group and 2**-weeks RSV treated groups.

| Variable | BD  group  (*n*=5) | HFD-control group  (*n*=5) | HFD-RSV2- treated group  (*n*=5) | | HFD-RSV20- treated group  (*n*=5) | |
| --- | --- | --- | --- | --- | --- | --- |
| Initial body weight (g) | 21.0±0.28 | 22.6±0.16 | 22.1±0.28 | (n.s.) | 22.0±0.31 | (n.s.) |
| Final body weight (g) | 31.2±0.91 | 43.4±3.18 | 42.9±2.10 | (n.s.) | 43.8±1.91 | (n.s.) |
| Water intake (ml/day/mouse) | 6.88±0.41 | 8.19±0.43 | 7.91±0.61 | (n.s.) | 8.02±0.41 | (n.s.) |
| Food Intake (g/day/mouse) | 2.61±0.53 | 2.69±0.43 | 2.69±0.79 | (n.s.) | 2.59±0.71 | (n.s.) |
| Liver weight (g) | 1.02±0.11 | 2.31±0.22 | 2.41±0.43 | (n.s.) | 2.38±0.51 | (n.s.) |
| White-fat weight (g) | 0.67±0.19 | 1.57±0.42 | 1.49±0.51 | (n.s.) | 1.45±0.48 | (n.s.) |
| HOMA-IR | 2.2±0.4 | 3.73±0.91 | 3.59±0.73 | (n.s.) | 3.65±0.11 | (n.s.) |

NAFL: nonalcoholic fatty liver, BD: basal diet, HFD: high-fat diet, RSV: resveratrol, RSV2: resveratrol 2mg/kg/day, RSV20: resveratrol 20mg/kg/day, HOMA-IR: homeostasis model for the assessment of insulin resistance. Data are the mean ± standard deviation; n.s., no significant difference *vs* HFD-control group (*n*=5 per group).

**Supplementary Table S2. Expressed gene sets in th**e livers of HFD-RSV20 mice compared with HFD-control mice (based on cDNA microarray analyses)

| **Gene set** | log (ratio) | Fold change |
| --- | --- | --- |
| **Glucose metabolism** |  |  |
| Slc2a2 (Glut2) | −0.0018 | 0.998753113 |
| PEPCK | −0.0454 | 0.96902111 |
| G6P | 0.033 | 1.02313747 |
| **Lipid metabolism** |  |  |
| SREBP1c | 0.0495 | 1.034906191 |
| Fasn | 0.0246 | 1.017197626 |
| **Mitochondrial β-oxidation of fatty acids** |  |  |
| PPARα | 0.033 | 1.02313747 |
| CPT-1a | 0.0769 | 1.054749205 |
| CPT-2 | 0.0201 | 1.014029765 |
| **VLDL synthesis** |  |  |
| MTTP | −0.0805 | 0.945729825 |
| ApoB | −0.0074 | 0.994883843 |
| **Insulin signalling** |  |  |
| IRS-1 | 0.0026 | 1.001803808 |
| IRS-2 | 0.3813 | 1.302515012 |
| FOXO1 | 0.0817 | 1.058264312 |
| **Histone deacetylase** |  |  |
| SIRT1 | −0.132 | 0.912565489 |
| **Heat production** |  |  |
| UCP1 | −0.0321 | 0.977995682 |
| UCP2 | 0.0395 | 1.027757571 |
| UCP3 | −0.0219 | 0.984934711 |
| **Innate immune response** |  |  |
| LBP | 0.0122 | 1.008492252 |
| CD14 | −1.1233 | 0.459042616* |
| TLR4 | 0.1504 | 1.109877153 |
| Myd88 | 0.7108 | 1.636711451 |
| IRAK1 | 0.0439 | 1.03089686 |
| IRAK4 | −0.164 | 0.892546971 |
| TRAF6 | −0.1094 | 0.926973499 |
| NF-κB | 0.5034 | 1.41755037 |
| **Nuclear receptor** |  |  |
| PPARγ | −0.0548 | 0.962727896 |
| **Cytokines** |  |  |
| TNF-α | −0.7778 | 0.583255536 |
| IL-6 | −0.069 | 0.953298545 |
| **Apoptosis** |  |  |
| Caspase-3 | −0.0009 | 0.999376362 |
| Caspase-9 | −0.1431 | 0.905571211 |
| **Fibrosis** |  |  |
| TGF-β | 0.147 | 1.107264584 |
| TIMP1 | −0.1039 | 0.930514154 |

Log ratios, fold change are based on high-fat diet (HFD)-resveratrol (RSV) 20 (RSV 20 mg/kg/day) mice (n=3) compared with HFD-control mice (n=3). *Hepatic CD14 mRNA showed greater fold-change compared with other genes.

ApoB, apolipoprotein B; CPT, carnitine palmitoyltransferase; Fasn, fatty acid synthase; FOXO, forkhead box O; Glut2, glucose transporter member 2; G6P, glucose-6-phosphatase; IL-6, interleukin-6; IRAK, interleukin-1 receptor-associated kinase; IRS, insulin receptor substrate; LBP, lipopolysaccharide binding protein; MTTP, microsomal triglyceride transfer protein; Myd88, myeloid differentiation primary response gene 88; NF-κB, nuclear factor-kappa B; PEPCK, phosphoenolpyruvate carboxykinase; PPAR, peroxisome proliferator activated receptor; Sirt, sirtuin; SREBP, sterol regulatory element-binding protein; TGF-β, transforming growth factor-beta; TIMP1, TIMP metallopeptidase inhibitor 1; TLR4, toll-like receptor 4; TNF-α, tumour necrosis factor α; TRAF-6, TNF receptor-associated factor 6; UCP, uncoupling protein.

**Supplementary Table S3. Nonalcoholic steatohepatitis (NASH) scoring system. Item, definitions, and scores used in this study.**

| Item | Definition | Score |
| --- | --- | --- |
| Steatosis | <5% | 0 |
|  | 5–33% | 1 |
|  | 33–66% | 2 |
|  | >66% | 3 |
| Lobular Inflammation | None | 0 |
|  | <2 foci/field | 1 |
|  | 2-4 foci/field | 2 |
|  | >5 foci/field | 3 |
| Hepatocyte ballooning | None | 0 |
|  | Few balloon cells | 1 |
|  | Many balloon cells | 2 |

**Supplementary Table S4. Fibrosis stage. Item, definitions, and stage used in this study.**

| Item | Definition | Stage |
| --- | --- | --- |
| Fibrosis | none  Isolated perisinusoidal or portal/periportal fibrosis | 0  1 |
|  | Perisinusoidal and portal/periportal fibrosis | 2 |
|  | Bridging fibrosis | 3 |
|  | Cirrhosis | 4 |

**Supplementary Table S5. Histological Scores of Livers using the NAFLD activity score (NAS) and fibrosis stage.**

| Item | BD+LPS | HFD+LPS- | HFD+LPS- | | HFD+LPS- | |
| --- | --- | --- | --- | --- | --- | --- |
|  | group | control group | RSV2- | | RSV20- | |
|  | (*n*=5) | (*n*=5) | treated group | | treated group | |
|  |  |  | (*n*=5) | | (*n*=5) | |
| Steatosis | 0 | 2.6±0.55 | 2.8±0.45 | (n.s) | 2.4±0.45 | (n.s) |
| Lobular inflammation | 0 | 2.4±0.45 | 1.4±0.55 | # | 1.2±0.45 | # |
| Hepatocyte ballooning | 0 | 2 | 1.4±0.55 | # | 1.2±0.45 | # |
| NAS | 0 | 6.8±0.84 | 5.4±0.55 | # | 5.0±0.70 | # |
| fibrosis stage | 0 | 2.4±0.55 | 1.4±0.55 | # | 1.2±0.0.45 | # |

NAFLD activity score (NAS) and fibrosis stage were scored according to the method described by Brunt and Kleiner et al. ^18, 19^, as outlined in Supplementary Table S3 and Supplementary Table S4. NAFLD: non-alcoholic fatty liver disease, NAS: non-alcoholic fatty liver disease activity score, BD: basal diet, HFD: high-fat diet, LPS: lipopolysaccharide, RSV: resveratrol, RSV2: resveratrol 2 mg/kg/day, RSV20: resveratrol 20 mg/kg/day. Data are the mean ± standard deviation. Significance was determined using the Mann–Whitney test for non-parametric factors. #*P*<0.05, significant difference *vs*. HFD+LPS-control group. (*n*=5 per group).

**Supplementary Methods**

***cDNA microarray analyses***

Total RNA (100 ng) was processed on microarrays using a GeneChip WT PLUS Reagent kit (Affymetrix, Santa Clara, CA, USA) according to manufacturer instructions. The resultant single-strand cDNA was fragmented and labelled with biotin, then hybridized to the Human (or Mouse) Gene 2.0 ST Array (Affymetrix). Arrays were washed, stained and imaged using a 450 Fluidics Station and GeneChip Scanner 3000 7G (Affymetrix) according to manufacturer instructions. Expression values were generated using Expression Console v1.3 (Affymetrix) with default robust multichip analysis parameters. Twofold change in gene expression was used as the threshold to denote an effect on expression.

***Immunohistochemistry***

Immunohistochemistry was undertaken on liver sections (thickness, 7µm). Primary antibody of neutrophil elastase (NE) was obtained from Abcam, F4/80 was obtained from eBioscience. Horseradish peroxidase-conjugated secondary antibody was purchased from Cell Signaling Technology. Deparaffinized sections were incubated in hydrogen peroxide/methanol. Endogenous avidin and biotin were blocked. Then 20% pig serum was added. Sections were incubated overnight with primary antibodies and developed and counterstain with Mayer’s haematoxylin the next day. NE slides at ×200 magnification and F4/80 slides at ×400 magnification were blinded and a minimum of five random fields were counted manually.

***Masson Trichrome staining***

Liver samples were excised and embedded in paraffin for histological analyses. Formalin-fixed and paraffin-embedded sections were processed routinely with Masson Trichrome (MT) staining. To quantify the fibrotic area of MT staining, images of five random fields of each section were processed with Photoshop Elements v13 (Adobe Systems, San Jose, CA, USA). Each value was expressed as the percentage of the total area of the section.
